# Effect duration of lumbar sympathetic ganglion neurolysis in patients with complex regional pain syndrome: a prospective observational study

**DOI:** 10.1038/s41598-024-63732-2

**Published:** 2024-06-03

**Authors:** Eun Joo Choi, Sunmin Kim, Dongsik Lim, Hyun Seung Jin, Sung Man Hong, Pyung Bok Lee, Francis Sahngun Nahm

**Affiliations:** 1 Department of Anesthesiology and Pain Medicine, Seoul National University Bundang Hospital, Seongnam, South Korea; 2 Department of Anesthesiology and Pain Medicine, Korea University Anam Hospital, Seoul, South Korea; 3 The Comfortable Pain Clinic, Seoul, South Korea; 4 Department of Anesthesiology and Pain Medicine, Dankook University College of Medicine, Cheonan, South Korea; 5 Department of Anesthesiology and Pain Medicine, Seoul National University College of Medicine, Seoul, South Korea

**Keywords:** Complex regional pain syndrome, Ganglia, Symathetic, Nerve block, Thermography, Diseases, Medical research, Neurology

## Abstract

Lumbar sympathetic ganglion neurolysis (LSGN) has been used for long-term pain relief in patients with complex regional pain syndrome (CRPS). However, the actual effect duration of LSGN has not been accurately measured. This prospective observational study measured the effect duration of LSGN in CRPS patients and investigated the relationship between temperature change and pain relief. After performing LSGN, the skin temperatures of both the maximum pain site and the plantar area in the affected and unaffected limbs were measured by infrared thermography, and pain intensity was assessed before and at 2 weeks, 1 month, and 3 months. The median time to return to baseline temperature was calculated using survival analysis. The skin temperature increased significantly at all-time points relative to baseline in both regions (maximum pain site: 1.4 °C ± 1.0 °C, plantar region: 1.28 °C ± 0.8 °C, all *P* < 0.001). The median time to return to baseline temperature was 12 weeks (95% confidence interval [CI] 7.7–16.3) at the maximum pain site and 12 weeks (95% CI 9.4–14.6) at the plantar area. Pain intensity decreased significantly relative to baseline, at all-time points after LSGN. In conclusion, the median duration of the LSGN is estimated to be 12 weeks.

## Introduction

Lumbar sympathetic ganglion block (LSGB) has been used in the treatment of peripheral vascular disease, hyperhidrosis, complex regional pain syndrome (CRPS), and other neuropathic pain conditions^1–3^. Because of its diagnostic and therapeutic significance, LSGB can be performed if a patient with CRPS is thought to have sympathetically mediated pain (SMP)^4^. It has been hypothesized that repeated afferent input from SMP in patients with CRPS exacerbates the hypersensitivity of alpha-adrenergic receptors in affected limbs, and abnormal chemical coupling may occur between the nociceptive and sympathetic systems^5,6^. Additionally, post-traumatic inflammation by repeated afferent input might be the first step in CRPS pathophysiology, and sympathetic activity can be a co-contributor to SMP in CRPS^7^. Also, thoracic sympathectomy is performed to reduce pain in the upper extremities caused by various diseases, including CRPS^8,9^.

If LSGB with local anesthetics shows only a short-term significant reduction in pain, repeated blocks or neurolysis (radiofrequency lesioning or chemical neurolysis) can be considered as modalities that are expected to exhibit long-term effects^10^. Despite variation in neurolytic agents and techniques, published studies have obtained broadly consistent outcomes, with 59%–72% of patients reporting alleviation of pain^11^.

The actual effect duration of lumbar sympathetic ganglion neurolysis (LSGN) has not been accurately measured. Few previous studies have reported assessments of pain relief after LSGN in patients with CRPS up to a defined period, which varied between 2 and 6 months^12,13^. However, those previous studies only measured the pain reduction in patients with CRPS over time, rather than skin temperature as an objective indicator. The success of LSGB or LSGN needs to be judged by comparing the skin-temperature increase of the affected lower limb with that of the unaffected limb^14^. The reason for this is that a rise in skin temperature is an objective sign of successful LSGB or LSGN, because sympathetic blocks cause vasodilation, which increases blood flow and temperature^4,15,16^.

LSGN is expected to have a longer effect than LSGB; however, no study has examined the duration of LSGN-related temperature increases of the affected limbs in CRPS as an objective indicator. Furthermore, previous studies on the effect duration of LSGN were all retrospective. Therefore, this study aimed to determine the duration of temperature rise in the affected limb after LSGN. We also examined the association between the temperature change and pain alleviation in the affected limb.

## Methods

### Study design and ethics approval

This prospective observational study was approved by the Institutional Review Board of Seoul National University Bundang Hospital (No. B-1508/312-308) and registered in the Clinical Research Information Service (https://cris.nih.go.kr; registration number KCT0001687). All study participants were informed of this study, and written informed consent was obtained. All the methods employed in this study were performed in accordance with the guidelines stipulated in the Declaration of Helsinki. We complied with the requirements established by the Consolidated Standards of Reporting Trials (CONSORT).

### Patients

The inclusion criteria were as follows: (1) age 18–60 years; (2) patients diagnosed with CRPS type 1 or 2 of the lower extremities based on the criteria of Harden et al. (Budapest criteria)^17^; (3) patients who had undergone LSGB at the L3 level twice before LSGN, followed by ≥ 50% pain relief; (4) patients with pain duration of ≤ 2 years after a painful event; (5) a baseline pain intensity score of ≥ 6 on the visual analogue scale (VAS, 0 = no pain, 10 = worst pain imaginable) after conservative treatment; and (6) patients in whom the affected extremity had a lower temperature (≥ 1 °C) than the unaffected side.

The exclusion criteria were as follows: (1) patient refusal; (2); patients who had not received LSGN after LSGB because LSGN was not necessary (sufficient pain relief after LSGB, as indicated by a VAS score ≤ 1) or LSGN was not considered to be beneficial (no effect after LSGB); (3) patients who had received LSGN or lumbar sympathetic heat radiofrequency ablation previously; (4) patients who were taking vasodilating agents; (5) patients who had concomitant disease that could contribute to another neuropathic pain condition; and (6) patients with a history of previous lumbar spine surgery.

### Procedures

One pain physician with > 10 years of expertise performed all procedures in an operating room under sterile conditions with fluoroscopic guidance. Each patient was placed on a radiographic table in the prone position with a pillow placed under the abdomen. After establishing an intravenous access line with Ringer’s lactate infusion, electrocardiography, automated blood pressure, and pulse oximetry were monitored. Temperature probes were attached to the center of both plantar surfaces to monitor the skin temperature during the procedure. After local anesthetic infiltration at the entry point, a 21-gauge 15-cm Chiba needle (Cook Inc., Bloomington, IN, USA) was advanced under fluoroscopic guidance until it reached the anterior margin of the L3 vertebral body on lateral view. To ensure that the needle was positioned correctly and to detect possible intramuscular or intravenous spread, 1–2 mL of contrast medium (Omnipaque®, Nycomed Ireland Ltd., Cork, Ireland) was administered. If the contrast agent did not spread properly, the procedure was repeated by inserting another needle at the L2 vertebral level. After verifying the final position of the needle tip, 5 mL of 0.25% ropivacaine was administered. A temperature increase ≥ 2 °C from the baseline within 20 min after injection of the drug was considered to indicate successful LSGB^18^. Patients revisited the outpatient pain center 1 week after the first LSGB procedure to receive the second procedure.

Whether or not to perform LSGN was determined 1 week after the second LSGB on the basis of the patient’s pain relief after the first and second LSGB. LSGN was implemented if the patient had a VAS score reduction > 50% relative to baseline.

The preparations for LSGN and LSGB were the same. After injecting 2.5 mL of 0.25% ropivacaine at the L2 and L3 levels, 2.5 mL of 99% ethanol was injected if there were no adverse effects 20 min later. Patients were kept prone for 1 h under close monitoring for any complications in the recovery room.

### Skin-temperature measurements

For skin-temperature measurements, infrared thermographs (IRTs) were performed five times. The baseline IRT was taken before the first LSGB, and a second IRT was taken on the procedure day immediately after the LSGN procedure. Following the LSGN procedure, IRT images were acquired at 2 weeks, 1 month, and 3 months, according to a standard protocol: each patient was acclimated in an isolated room at a mean temperature of 23 °C and a relative humidity of 50% for 20 min with no clothing. A digital infrared thermal imaging system (T-1000 Smart®, Mesh Co., Ltd., Wonju, Korea), which consisted of a computer-assisted infrared camera and a display module, was used to obtain the IRT images. The IRT camera was positioned 1 m from the patient, and in the standing position, IRT images of the front, back, and both sides of the lower extremities and the feet of the patient were captured.

The temperature difference between the affected and unaffected lower extremities was measured. After determining the regions of interest (ROIs) as the patient’s maximum pain site and the plantar area, the temperature differences (∆T) in the maximum pain site and the plantar area were calculated according to the following equation.

ΔT = skin temperature of the ROIs in the affected limb—skin temperature of the ROIs in the unaffected limb.

**Figure 1:**
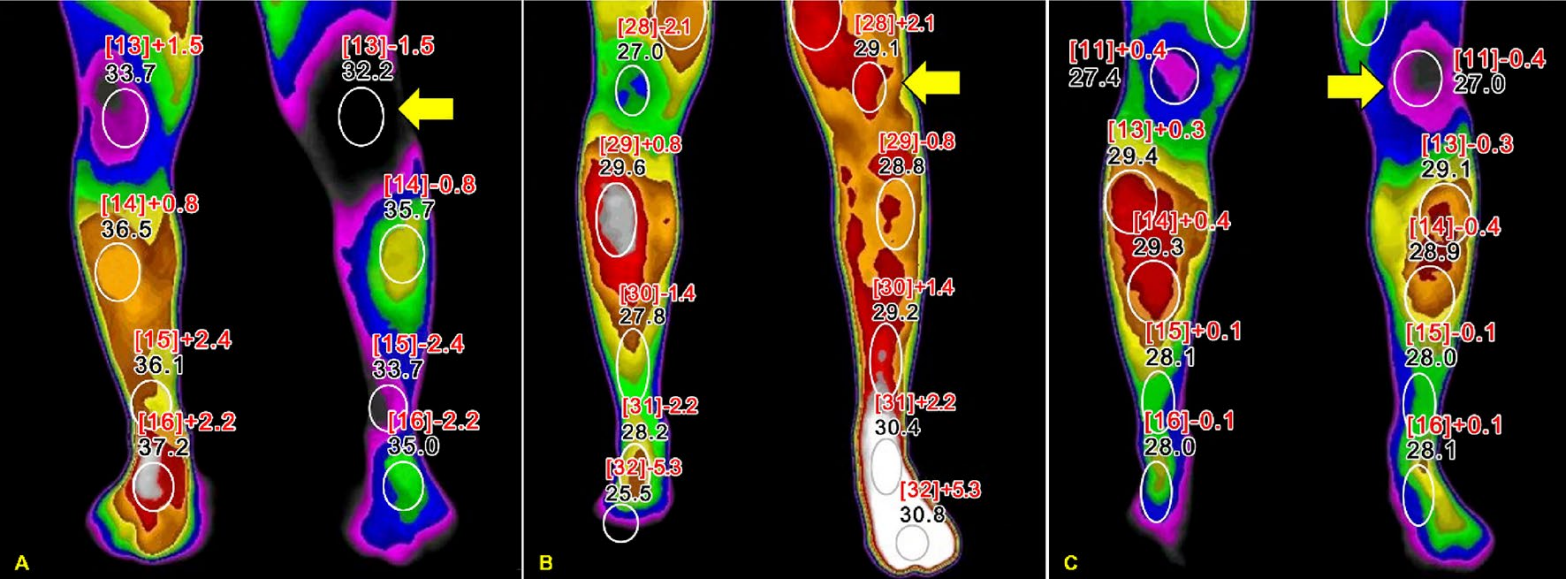
Infrared thermographic images of a 26-year-old patient with complex regional pain syndrome type 1, showing results in the lower extremities. The maximum pain site of this patient was the left knee (yellow arrows). The skin temperature differences between the affected and unaffected site (∆T) at three time points are illustrated. (**A**) Baseline ∆T = − 1.5 °C; (**B**) ∆T immediately after lumbar sympathetic neurolysis = 2.1 °C; (**C**) ∆T two weeks after LSGN =  − 0.4 °C. The numbers in the square brackets indicates the numbers of the region of interest.

The IRTs from pre-LSGN (baseline), post-LSGN, 2 weeks, 1 month, and 3 months following the procedure were analyzed by an independent physician. The baseline was the value of the first IRT before the first LSGB was performed (Fig. 1). The temperature difference at the maximum pain site and the plantar area was recorded at each time point. The patients did not undergo any other interventional procedures until 3 months after LSGN.

### Evaluation of clinical effectiveness

We used the VAS score to measure the pain intensity in the affected lower extremity before the first LSGB and at 2 weeks, 1 month, and 3 months after LSGN. Additionally, we collected the following data for each patient before the first LSGB: age, sex, height, weight, symptom duration, maximum pain site location in the affected lower extremity, and information on medications being taken, such as strong opioid analgesics (e.g., oxycodone, hydromorphone, morphine, or transdermal fentanyl), non-opioid analgesics (e.g., tramadol, nonsteroidal anti-inflammatory drugs, or acetaminophen), anticonvulsants (e.g., pregabalin or gabapentin), and antidepressants. We also noted the type and cause of CRPS (trauma, surgery, fracture, or combined) and any coexisting psychopathology.

### Statistical analysis

The primary outcome variable of this study was the ∆T at 3 months after LSGN. Because there has been no prospective study on LSGN, we calculated the required sample size from the following conditions: Cohen’s f = 0.25 (medium effect size), α = 0.05, β = 0.8, number of groups = 1, repetitions = 5, correlation among repeated measures = 0.5, and nonsphericity correction ε = 1^19^. The minimum sample size needed was 20 patients. Allowing for a drop-out rate of 20%, the required sample size was 25 patients. The sample size was calculated using G*Power version 3.1.0 (Heinrich Heine University, Düsseldorf, Germany). Friedman’s test, followed by Dunnet’s post hoc analysis, was used to compare the difference in ∆T at each time point. Furthermore, Kaplan–Meier survival analysis was performed to estimate the LSGN effect duration. MedCalc® statistical software version 20.218 (MedCalc Software Ltd., Ostend, Belgium) and IBM SPSS Statistics version 28.0 (IBM Corp., Armonk, NY, USA) were used for statistical analyses. Statistical significance was defined as a *P* value of < 0.05.

## Results

**Figure 2:**
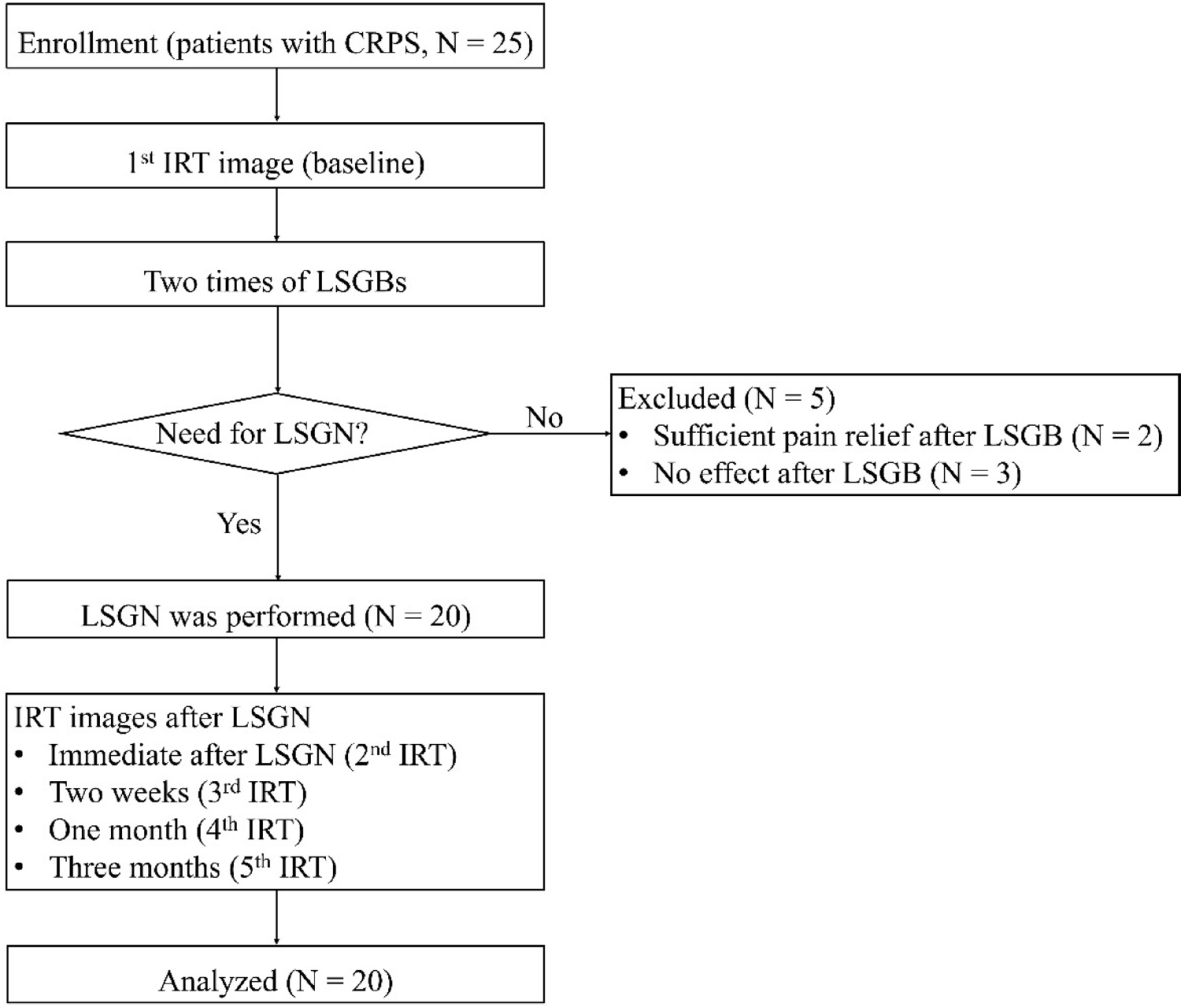
CONSORT diagram of the patients included in this study. *CRPS* complex regional pain syndrome, *LSGB* lumbar sympathetic ganglion block, *LSGN* lumbar sympathetic ganglion neurolysis.

Twenty-five patients were enrolled in this study. Five patients were excluded after undergoing LSGB twice. Two patients had substantially improved pain and another had aggravated pain after their procedures. Finally, 20 patients received LSGN, and all of them completed the study (Fig. 2). The patients’ characteristics are shown in Table 1.

**Table 1:** Demographic and clinical characteristics of the patients.

Variable	Data
Age (years)	38.5 ± 10.6
Height (cm)	167.9 ± 6.9
Weight (kg)	70.9 ± 7.6
Pain duration (weeks)	31.3 ± 13.8
Sex (male/female)	13/7
CRPS type 1/2	18/2
Cause of pain (n)	
Trauma	11
Operation	6
Both	3
Psychopathology (yes/no)	13/7
Maximal pain site	
Thigh	3
Calf	1
Ankle	11
Foot	5
Medications (yes/no)	
Strong opioids	6/14
Weak opioids	20/0
Anticonvulsant	20/0
Antidepressant	20/0

Values are presented as the mean ± standard deviation or numbers.

*CRPS* complex regional pain syndrome.

**Figure 3:**
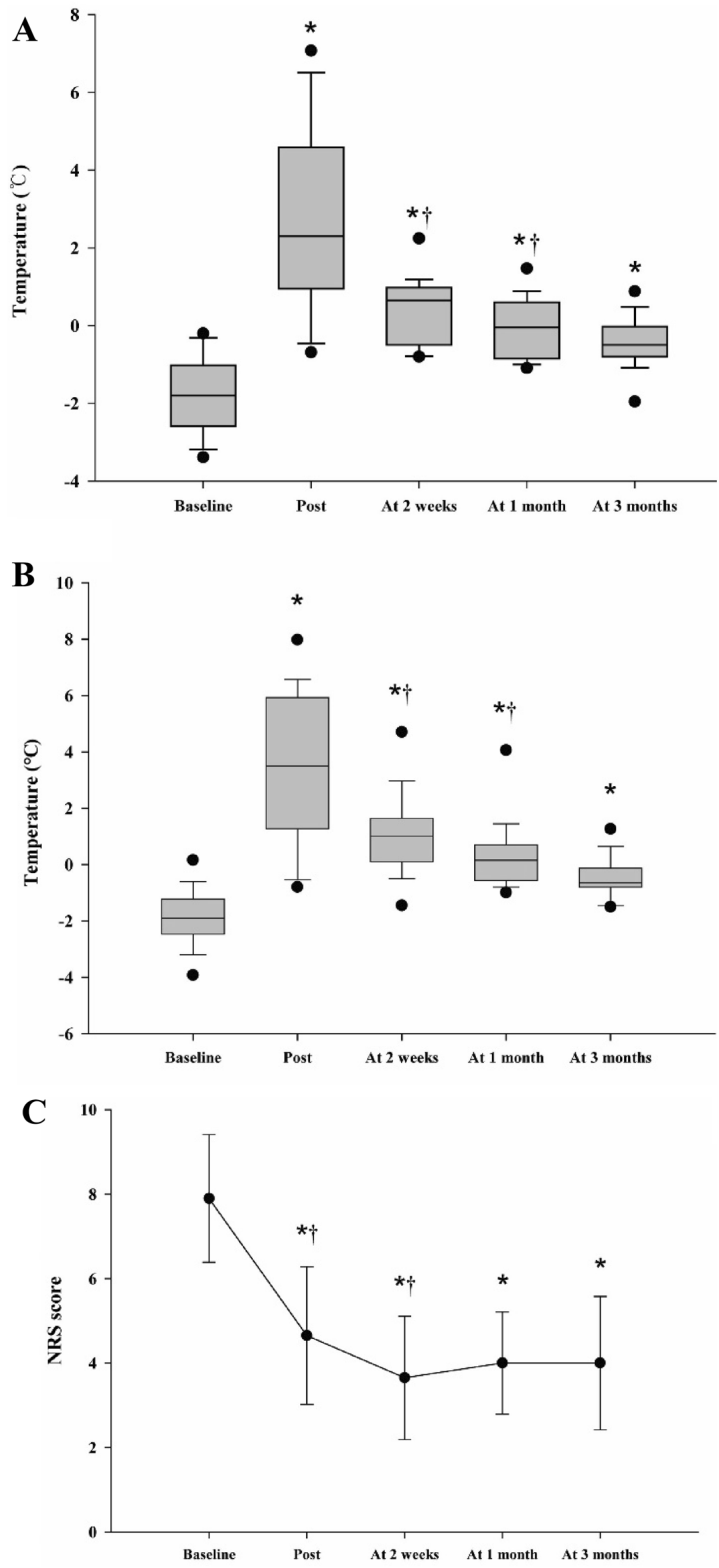
Skin-temperature change at the maximum pain site (**A**) and plantar area (**B**) and the patients’ pain intensity (**C**) pre-LSGN (baseline), post-LSGN, and 2 weeks, 1 month, and 3 months after LSGN (*P < 0.001 compared with pre-LSGN, †*P* < 0.001 compared with the previous time point). There was a significant increase in temperature in both regions and a decrease in the patient’s pain at all time points relative to baseline. *LSGN* lumbar sympathetic neurolysis.

The changes in ΔT in both regions (maximum pain site and plantar region) of the patients are shown in Figs. 3A,B. The mean temperature rise was 4.9 °C ± 0.4 °C at the maximum pain site and 5.4 °C ± 0.6 °C at the planter region immediately after LSGN. The increased temperature gradually decreased, but remained higher than the baseline temperature. The temperature significantly increased at all time points relative to the baseline in both regions (maximum pain site: 1.4 °C ± 1.0 °C, plantar region: 1.28 °C ± 0.8 °C, *P* < 0.001). Furthermore, we observed a significant decrease in pain intensity compared to baseline at all time points at the maximum pain site (Fig. 3C) (*P* < 0.001).

**Figure 4:**
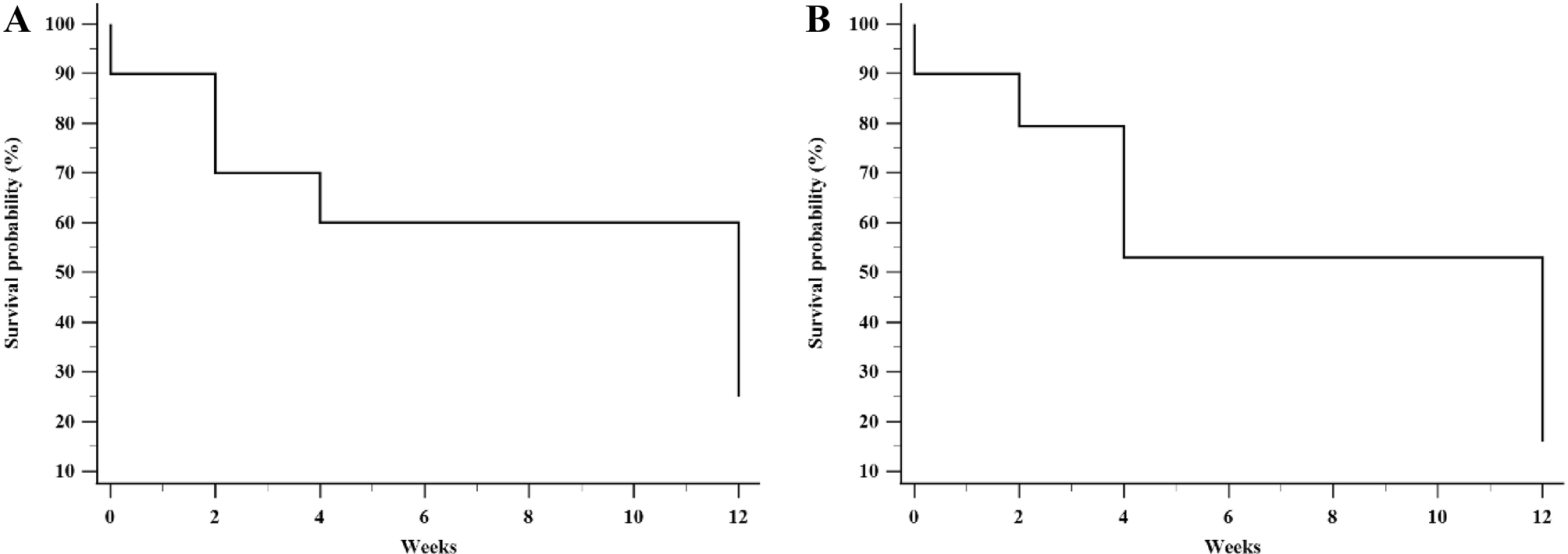
Kaplan–Meier survival analysis of the effect duration of lumbar sympathetic neurolysis. The median time to return to baseline temperature was 12 weeks (95% confidence interval [CI], 7.6–16.3 weeks) at the maximum pain site (**A**) and 12 weeks (95% CI 9.4–14.6 weeks) in the plantar area (**B**).

The pain intensity decreased significantly at all time points after LSGN relative to baseline. The median time to return to the baseline temperature was 12 weeks (95% confidence interval [CI], 7.7–16.3 weeks) for the maximum pain site and 12 weeks (95% CI 9.4–14.6 weeks) for the plantar area (Fig. 4A,B).

## Discussion

In this study, the temperature at the maximum pain site and the plantar surface increased after LSGN in all patients with CRPS. The temperature remained higher in both regions at 3 months after LSGN than at the baseline. Furthermore, the pain intensity at the maximum pain site decreased at all-time points after LSGN for the patients with CRPS. This is the first prospective study to observe the effect duration of LSGN by measuring the temperature in patients with CRPS. Several studies are consistent with our findings, implying that alcohol-induced sympatholysis lasts for at least 12 weeks. An investigation involving 386 patients experiencing rest pain attributable to occlusive vascular disease, treated with LSGN, found that the mean time for sweat test alteration was 6.0 months, with the duration of pain relief averaging 5.9 months. Notably, the initiation of pain relief corresponded with the occurrence of sympathetic nerve blockade and the subsequent augmentation of cutaneous blood flow^20^. Furthermore, several previous studies have reported that celiac plexus neurolysis with alcohol to treat abdominal pain in cancer patients reduced pain by 11–14 weeks^21–23^. Neurolysis with alcohol disrupted pain signals for 3–6 months, possibly through nonselective destruction of nervous tissue by denaturing proteins and fatty substance extraction^24^. This finding might support the link between the length of pain relief and the sign of sympathetic blockage.

LSGN can be considered for patients with CRPS and SMP, and its long-term effect is also important. Only one study has prospectively measured the duration of the blocking effect after LSGN through an objective test (sweat test and temperature measurement), comparing the effectiveness of phenol neurolysis (n = 8) with that of a heated radiofrequency (n = 9) on the lumbar sympathetic ganglion in CRPS patients^25^. In the LSGN group treated with phenol, 89% of patients exhibited signs of sympathetic block up to 8 weeks following the procedure. However, the study did not measure pain relief after LSGN, and therefore could not establish a correlation between the sympathetic block effect and pain relief.

Some retrospective studies have found no correlation between pain relief following a sympathetic block and temperature increase in patients with CRPS. These studies examined the relationship between temperature increase and pain reduction immediately after a single LSGB, stellate ganglion block, or LSGN in patients with CRPS^12,26^. The results indicated a very weak correlation between temperature rise and immediate pain relief following the procedure, despite patients with larger temperature increases experiencing greater immediate pain relief. Furthermore, no correlation was found between the effect of the sympathetic block and pain relief 4–8 weeks post-procedure^26^.

In our study, CRPS patients experienced a persistent temperature increase for up to 12 weeks following LSGN, and changes in skin temperature and pain reduction showed an inverse pattern. However, we were unable to statistically establish a clear correlation between these two factors. Previous studies have attempted to prove the correlation between the degree of sympathetic block and pain relief in CRPS patients but have not provided conclusive results. One reason could be that not all pain associated with CRPS is solely dependent on SMP.

In previous studies, 28–31% of patients with CRPS showed a good response to LSGN or LSGB, indicating that their pain was SMP^12,15^. Although activation of the sympathetic nervous system does not normally aggravate pain, the pathophysiology of SMP has been proposed as an important mechanism of pain in CRPS. SMP is related to peripheral neuroinflammation, mostly during early-phase CRPS^7^. Limb injury or trauma triggers local inflammation, and it can upregulate α1-adrenoceptors in keratinocytes and nociceptors^27^. Activation of the sympathetic nervous system induces the release of adrenaline, which activates upregulated α1-adrenoceptors, resulting in severe pain and hyperalgesia in the affected lesion. However, as the disease progresses in CRPS, the role of sympathetic activity in pain appears to decrease^28^, which would support the proposal that SMP may be more prevalent in early CRPS. In our study, the mean duration of symptoms was 31.3 ± 13.8 weeks; thus, patients with early-phase CRPS were included. It is plausible that patients with early-phase CRPS and SMP responded well to the sympathetic block and experienced a greater reduction in pain after LSGN. Furthermore, a previous study reported that early sympathetic block improved the treatment effect on neuropathic pain in extremities^29^. Sympathetic block was more effective in patients with symptom durations of < 1 year, whereas other factors did not show a significant relationships with treatment effects. Sympathetic block was more effective in patients with symptom durations of less than 1 year, while other factors did not show a correlation with treatment effects. In our findings, the median time for the maximum pain site to return to baseline temperature was 12 weeks, and the improvement in pain also decreased during this period. In our study, CRPS patients demonstrated sufficient effects from LSGB and pain reduction over a period of 12 weeks after LSGN, suggesting that they may be suffering from SMP-related CRPS. However, even in patients with CRPS, their pain is not solely due to SMP, implying that there might be multi-dimensional aspect of pain in CRPS other than SMP. Therefore, it is likely that we were unable to establish a direct correlation between the effects of sympathetic blocks and pain reduction solely based on sympathetic block effects. Because the treatment for CRPS may be insufficient with only sympathetic blocks, and it requires a multimodal approach^30^. In addition to sympathetic blocks, pharmacological agents, rehabilitation interventions, pain exposure therapy, neuromodulation, and intrathecal morphine or baclofen pump are being attempted for relieving pain in CRPS, but so far, they only provide uncertain evidence^31,32^. Also, Harden, et al. pointed out in a recent review that due to CRPS being a rare and complicated multifactorial disease, previous studies clearly demonstrating treatment efficacy are limited^33^. Therefore, in our study, other factors in CRPS patients that either exacerbate or alleviate pain (not associated with SMP) may have influenced the treatment outcomes, which might explain why we were unable to establish a direct correlation between sympathetic nervous system activity and pain.

In this study, IRT was utilized as a non-contact method to visualize skin temperature. Several studies have established the validity of IRT in diagnosing CRPS^6,34,35^. To ensure an accurate evaluation of the response after LSGN, we chose to measure the response not only at the site of the patients’ most severe pain in the lower extremity, but also at the plantar surface.

The present study had several limitations. First, it was not feasible to conduct follow-up beyond the 12-week period after LSGN because patients’ agonizing pain often precluded further observation without another intervention in the clinical setting. A study observing the effects of LSGN for a longer time is needed in the future. Second, because there was no control group receiving conservative treatment, we could not differentiate the effect of LSGN from the natural course of patients with CRPS. Third, although changes in skin temperature and pain reduction after LSGN showed an inverse pattern, we could not establish a statistical association between these two factors. The reason may be that CRPS is a disease with a multifactorial pathophysiology not solely associated with SMP. Fourth, our study design involved pre-selecting participants who had positive responses to long-acting splanchnic nerve blocks (LSGB) before undergoing long-acting splanchnic neurolysis (LSGN). This pre-selection could introduce potential participant recruitment bias. However, given the high-risk nature of LSGN involving chemical neurolysis, a preliminary screening procedure using the lower-risk LSGB with local anesthetics is a necessary precaution. This approach follows established practices documented in prior research where LSGB was performed before progressing to LSGN. Furthermore, the consecutive recruitment strategy employed in this study helps minimize the potential for selection bias^12,13^. Nevertheless, this study has value as the first research to determine the effect duration of LSGN, by measuring both objective (temperature) and subjective (pain) variables in patients with CRPS.

## Conclusions

The effect of LSGN on reducing pain and increasing temperature in the affected extremity was sustained for ≤ 12 weeks post-treatment, with a significant reduction in pain intensity after LSGN. These results support the use of LSGN as a prolonged pain management strategy in patients with CRPS.

## Data Availability

The datasets supporting the findings of this study are available from the corresponding author upon reasonable request.

## References

[1] Boas RA (1998). Sympathetic nerve blocks: In search of a role. Reg. Anesth. Pain Med..

[2] Wu Y (2022). Predictive value of contrast-enhanced ultrasound in chemical lumbar sympathectomy for end-stage arteriosclerosis obliterans of the lower extremities. Pain Ther..

[3] Zhu X, Kohan LR, Morris JD, Hamill-Ruth RJ (2019). Sympathetic blocks for complex regional pain syndrome: A survey of pain physicians. Reg. Anesth. Pain Med..

[4] Krumova EK (2011). Are sympathetic blocks useful for diagnostic purposes?. Reg. Anesth. Pain Med..

[5] van Eijs F (2011). Evidence-based interventional pain medicine according to clinical diagnoses. 16. Complex regional pain syndrome. Pain Pract..

[6] Bruehl S (2010). An update on the pathophysiology of complex regional pain syndrome. Anesthesiology.

[7] Knudsen LF, Terkelsen AJ, Drummond PD, Birklein F (2019). Complex regional pain syndrome: A focus on the autonomic nervous system. Clin. Auton. Res..

[8] Hashmonai M, Cameron AE, Licht PB, Hensman C, Schick CH (2016). Thoracic sympathectomy: A review of current indications. Surg. Endosc..

[9] Rocha Rde O (2014). Thoracic sympathetic block for the treatment of complex regional pain syndrome type I: A double-blind randomized controlled study. Pain.

[10] Zacharias NA, Karri J, Garcia C, Lachman LK, Abd-Elsayed A (2021). Interventional radiofrequency treatment for the sympathetic nervous system: A review article. Pain Ther..

[11] Zechlinski JJ, Hieb RA (2016). Lumbar sympathetic neurolysis: How to and when to use?. Tech. Vasc. Interv. Radiol..

[12] Dev S (2018). Does temperature increase by sympathetic neurolysis improve pain in complex regional pain syndrome? A retrospective cohort study. World Neurosurg..

[13] Manjunath PS, Jayalakshmi TS, Dureja GP, Prevost AT (2008). Management of lower limb complex regional pain syndrome type 1: An evaluation of percutaneous radiofrequency thermal lumbar sympathectomy versus phenol lumbar sympathetic neurolysis–a pilot study. Anesth. Analg..

[14] Kim YC, Bahk JH, Lee SC, Lee YW (2003). Infrared thermographic imaging in the assessment of successful block on lumbar sympathetic ganglion. Yonsei Med. J..

[15] van Eijs F (2012). Predictors of pain relieving response to sympathetic blockade in complex regional pain syndrome type 1. Anesthesiology.

[16] Feigl GC (2014). Susceptibility of the genitofemoral and lateral femoral cutaneous nerves to complications from lumbar sympathetic blocks: Is there a morphological reason?. Br. J. Anaesth..

[17] Harden RN, Bruehl S, Stanton-Hicks M, Wilson PR (2007). Proposed new diagnostic criteria for complex regional pain syndrome. Pain Med..

[18] Greenstein D, Brown TF, Kester RC (1994). Assessment of chemical lumbar sympathectomy in critical limb ischaemia using thermal imaging. Int. J. Clin. Monit. Comput..

[19] Cohen, J. in *Statistical power analysis for the behavioural sciences.* 273–406 (Lawrence Erlbaum Associates, 1988).

[20] Cousins MJ, Reeve TS, Glynn CJ, Walsh JA, Cherry DA (1979). Neurolytic lumbar sympathetic blockade: Duration of denervation and relief of rest pain. Anaesth. Intensive Care.

[21] Ishiwatari H (2014). Phenol-based endoscopic ultrasound-guided celiac plexus neurolysis for East Asian alcohol-intolerant upper gastrointestinal cancer patients: A pilot study. World J. Gastroenterol..

[22] Leblanc JK (2013). Endoscopic ultrasound-guided celiac plexus neurolysis in pancreatic cancer: A prospective pilot study of safety using 10 mL versus 20 mL alcohol. Diagn. Ther. Endosc..

[23] LeBlanc JK (2011). A prospective, randomized study of EUS-guided celiac plexus neurolysis for pancreatic cancer: One injection or two?. Gastrointest. Endosc..

[24] Choi EJ (2016). Neural ablation and regeneration in pain practice. Korean J. Pain.

[25] Haynsworth RF, Noe CE (1991). Percutaneous lumbar sympathectomy: A comparison of radiofrequency denervation versus phenol neurolysis. Anesthesiology.

[26] Samen CDK (2022). Correlation between temperature rise after sympathetic block and pain relief in patients with complex regional pain syndrome. Pain Med..

[27] Finch PM, Drummond ES, Dawson LF, Phillips JK, Drummond PD (2014). Up-regulation of cutaneous α1 -adrenoceptors in complex regional pain syndrome type I. Pain Med..

[28] Schattschneider J, Binder A, Siebrecht D, Wasner G, Baron R (2006). Complex regional pain syndromes: The influence of cutaneous and deep somatic sympathetic innervation on pain. Clin. J. Pain.

[29] Yoo HS, Nahm FS, Lee PB, Lee CJ (2011). Early thoracic sympathetic block improves the treatment effect for upper extremity neuropathic pain. Anesth. Analg..

[30] Shim H, Rose J, Halle S, Shekane P (2019). Complex regional pain syndrome: A narrative review for the practising clinician. Br. J. Anaesth..

[31] Shafiee E (2023). The effectiveness of rehabilitation interventions on pain and disability for complex regional pain syndrome: A systematic review and meta-analysis. Clin. J. Pain.

[32] Limerick G (2023). Complex regional pain syndrome: Evidence-based advances in concepts and treatments. Curr. Pain Headache Rep..

[33] Harden, R. N. *et al.* Complex Regional Pain Syndrome: Practical Diagnostic and Treatment Guidelines, 5th Edition. *Pain Med*. **23**, S1-S53 (2022). 10.1093/pm/pnac046 PMC9186375 35687369

[34] Jeon SG (2014). Do severity score and skin temperature asymmetry correlate with the subjective pain score in the patients with complex regional pain syndrome?. Korean J. Pain.

[35] Schürmann M (2007). Imaging in early posttraumatic complex regional pain syndrome: A comparison of diagnostic methods. Clin. J. Pain.

